# Automated electronic medical record sepsis detection in the emergency department

**DOI:** 10.7717/peerj.343

**Published:** 2014-04-10

**Authors:** Su Q. Nguyen, Edwin Mwakalindile, James S. Booth, Vicki Hogan, Jordan Morgan, Charles T. Prickett, John P. Donnelly, Henry E. Wang

**Affiliations:** 1 University of Alabama School of Medicine, USA; 2 Department of Emergency Medicine, University of Alabama School of Medicine, Birmingham, AL, USA; 3 Department of Nursing Informatics, University of Alabama at Birmingham Hospital, Birmingham, AL, USA

**Keywords:** Sepsis, Automation, Automated alerts, Accuracy, Predictive value

## Abstract

**Background.** While often first treated in the emergency department (ED), identification of sepsis is difficult. Electronic medical record (EMR) clinical decision tools offer a novel strategy for identifying patients with sepsis. The objective of this study was to test the accuracy of an EMR-based, automated sepsis identification system.

**Methods.** We tested an EMR-based sepsis identification tool at a major academic, urban ED with 64,000 annual visits. The EMR system collected vital sign and laboratory test information on all ED patients, triggering a “sepsis alert” for those with ≥2 SIRS (systemic inflammatory response syndrome) criteria (fever, tachycardia, tachypnea, leukocytosis) plus ≥1 major organ dysfunction (SBP ≤ 90 mm Hg, lactic acid ≥2.0 mg/dL). We confirmed the presence of sepsis through manual review of physician, nursing, and laboratory records. We also reviewed a random selection of ED cases that did not trigger a sepsis alert. We evaluated the diagnostic accuracy of the sepsis identification tool.

**Results.** From January 1 through March 31, 2012, there were 795 automated sepsis alerts. We randomly selected 300 cases without a sepsis alert from the same period. The true prevalence of sepsis was 355/795 (44.7%) among alerts and 0/300 (0%) among non-alerts. The positive predictive value of the sepsis alert was 44.7% (95% CI [41.2–48.2%]). Pneumonia and respiratory infections (38%) and urinary tract infection (32.7%) were the most common infections among the 355 patients with true sepsis (true positives). Among false-positive sepsis alerts, the most common medical conditions were gastrointestinal (26.1%), traumatic (25.7%), and cardiovascular (20.0%) conditions. Rates of hospital admission were: true-positive sepsis alert 91.0%, false-positive alert 83.0%, no sepsis alert 5.7%.

**Conclusions.** This ED EMR-based automated sepsis identification system was able to detect cases with sepsis. Automated EMR-based detection may provide a viable strategy for identifying sepsis in the ED.

## Introduction

Sepsis is the syndrome of microbial infection complicated by systematic inflammation which may subsequently lead to organ dysfunction, shock, and death (Levy et al., 2003). Sepsis is a major public health problem, accounting for more than 750,000 hospital admissions, 500,000 emergency department (ED) visits and 200,000 deaths annually (Angus et al., 2001; Annane, Bellissant & Cavaillon, 2005; Jones, 2006). Early aggressive therapy is essential for optimizing outcomes from sepsis (Rivers et al., 2001).

In recent years, physicians have increasingly utilized electronic medical records (EMR) systems to aid clinical decision making (Levy & Heyes, 2012). By collecting and organizing clinical data, EMR systems have strong potential to improve the detection of conditions where symptoms or laboratory findings are difficult to discern. Diagnosis of sepsis is difficult because clinicians may not recognize the constellation of clinical, physiologic and laboratory abnormalities that comprise the syndrome. Several efforts have attempted to use EMR systems for sepsis detection, albeit with marginal results (Jaimes et al., 2003; Nelson et al., 2011). A prominent limitation of these prior efforts was the absence of data for hypotension or lactic acidosis, which are often prominent features of sepsis and may indicate the need for aggressive protocolized resuscitation (Rivers et al., 2001).

In this study we sought to evaluate the accuracy of an automated EMR sepsis detection system in the ED.

## Methods

### Study design

We conducted a retrospective analysis of automated clinical data collected by an ED EMR system. The study was approved via a written application by the Institutional Review Board of the University of Alabama at Birmingham (approval #X120409014).

### Study setting

This study utilized ED data from the University of Alabama at Birmingham (UAB) Hospital, an urban academic tertiary care referral medical center in Birmingham, Alabama, United States. The ED treats over 64,000 patients annually and is the only Level I trauma center in Alabama. While the ED does not restrict the age of treated patients, the ED population is predominantly (>99%) adult. UAB Hospital has over 900 inpatient beds, including more than 180 critical care beds.

### EMR sepsis detection system

The ED utilized the Cerner FirstNet^®^ (Kansas City, Missouri) EMR system. The FirstNet system collects comprehensive demographic and clinical information for all patients presenting receiving care in the ED, including patient demographics, location and status in the ED, care time points, laboratory and other test results, nursing and physician documentation, and patient education. Access to the master database is facilitated using a proprietary database language (Cerner Command Language) patterned after Structured Query Language (SQL).

Using the Cerner FirstNet platform we developed an automated sepsis detection system. The sepsis detection system drew upon clinical and laboratory information documented on all ED patients. The detection system was developed using the Cerner Discern Analytics^®^ v.2.0 reporting and data analysis tool, a Java-based program which is integrated with the EMR system.

The sepsis detection system triggered a “sepsis alert” if the EMR identified two or more Systemic Inflammatory Response Syndrome (SIRS) criteria and at least one sign of shock. SIRS criteria included (1) temperature ≤36 °C (96.8 °F) or ≥38 °C (100.4 °F), (2) respiratory rate ≥20 breaths/min, (3) heart rate ≥90 beats/min, and (4) total white blood cell (WBC) count ≤4,000 or ≥12,000 cells/mm^3^, or >10% bands. Signs of shock included (1) systolic blood pressure ≤90 mm Hg, or lactic acid ≥2.0 mg/dL. We chose these definitions based upon criteria used by Rivers et al. (2001) in a clinical trial of septic shock. While Rivers et al. (2001) used a lactic acid lactate threshold ≥4.0 mg/dL, we lowered this criterion to 2.0 mg/dL because from our clinical experience, many clinically septic patients presented with lactic acid levels in this range.

The EMR system generated sepsis alerts in real time as soon as combinations of findings fulfilled defined criteria. The system assessed data elements asynchronously; combinations of values from differing time points could be combined to activate an alert. Each fulfillment of additional sepsis criteria would result in the repeat activation of a sepsis alert. Vital signs were based upon nursing assessments entered into the EMR system. The hospital laboratory computer system (HealthQuest Data Systems, Highland, California) provided all laboratory test results.

While children have different ranges for SIRS criteria, <1% of ED patients were <18 years old (Goldstein, Giroir & Randolph, 2005). Therefore, we did not modify the sepsis alert rules by patient age.

### Selection of subjects

Automated sepsis screening occurred for all ED patients. The data for this study originated from a 3-month pilot testing period January 3, 2012 to March 31, 2012. During this period automated alerts were generated and evaluated *post hoc*, but were not communicated to clinicians.

Determination of the true diagnostic accuracy of the sepsis alert system would require manual review of ED records for all patients that did not activate the sepsis detection system. However, this would require manually reviewing over 18,000 ED medical records, which was not logistically feasible. In the effort to provide some comparison between sepsis alert and non-alert patients, we randomly selected 300 patients treated in the ED during the study period but who did not activate the EMR sepsis detection system. We chose this number based upon the availability of resources for manual medical record review.

### Outcomes—confirmation of sepsis

To confirm the presence or absence of sepsis in each ED patient, two investigators manually reviewed the ED medical records for all sepsis alert activations and the randomly selected non-alert cases. We defined sepsis as the presence of (1) a serious infection related to the ED presentation, (2) ≥2 SIRS criteria, and (3) systolic blood pressure ≤90 mm Hg or lactic acid level ≥2.0 mg/dL. We used previously published criteria to classify an infection as a “serious infection” (Angus et al., 2001; Wang et al., 2007). The presence of a serious infection was based upon ED clinician documentation, including the clinical narrative as well as ED diagnoses. We did not use laboratory or radiologic test results to confirm the presence of an infection. Because of our focus on the ED presentation, course of care, and clinical impression, we did not use medical records from later points of hospitalization nor discharge diagnoses to determine the presence of an infection. As part of the chart review process, the reviewers also confirmed the fulfillment of SIRS criteria by each automated alert. Therefore, reviewers were not blinded to presence or absence of a sepsis alert activation.

The reviewers resolved all discrepancies by consensus. In a test series of 30 records, inter-rater agreement for the presence of sepsis was high (kappa = 0.78).

### Data analysis

We determined the diagnostic accuracy of the automated EMR sepsis detection system by calculating positive predictive value (PPV) of the sepsis alerts. We estimated the negative predictive value based upon the sample of non-sepsis alert patients. Because of the sampled nature of the non-alerts, it was not possible to calculate the sensitivity, specificity and area under the ROC curve. We identified the infection category for true-positive sepsis alerts. We determined the chief reason for ED visit for false-positive sepsis alerts and true-negative non-sepsis alerts. We also determined the disposition of each patient (admitted to hospital, died in ED or discharged home from ED). We conducted all analyses using Stata v.12.2 (Stata, Inc., College Station, TX).

## Results

During the three-month study period, there were activations of the EMR sepsis alert system for 795 ED patients. The mean age of sepsis alert patients was 55 ± 20 years, and half were male (51%). There was a total of 1,224 alerts across the 795 patients. The median number of alerts was 2 per patient (IQR 1-2). The maximum number of alerts for an individual patient was 6.

Of the 795 EMR sepsis alerts, manual record review confirmed the presence of sepsis in 355 cases (Table 1). The positive predictive value of the sepsis alert system was 44.7% (95% CI [41.2–48.2%]). Among true-positive sepsis alerts, the most common infections were those of the respiratory and urinary tract (Table 2). Among the false-positive sepsis alerts, trauma, non-infectious gastrointestinal disorders and cardiovascular disorders were the most common conditions (Table 3).

**Table 1:** Emergency department (ED) automated sepsis alerts, January 1, 2012–March 31, 2012. Includes 795 ED visits with triggered sepsis alert. The table includes comparison with 300 randomly selected ED patients that did not trigger a sepsis alert. Positive predictive value of sepsis alert is 44.7% (95% CI [41.2–48.2%]).

Sepsis alert	Confirmed sepsis		
	Sepsis	No sepsis	Total
Yes	355	440	795
No	0	300	300
Total	293	802	1,095

**Table 2:** Infection types of emergency department visits with triggered sepsis alert and confirmed sepsis (true positive alert). Total of *n* = 355 true positive sepsis alerts. A patient may have had more than one infection.

Infection type	*n* (%)
Pneumonia or other respiratory	135 (38.0)
Urinary tract	116 (32.7)
Gastrointestinal	54 (15.2)
Bacteremia	49 (13.8)
Cellulitis	33 (9.3)
Abscess	26 (7.3)
Gynecologic	5 (1.4)
Central nervous system	3 (0.9)
Other infection	12 (3.4)

**Table 3:** Medical conditions of emergency department visits with triggered sepsis alert but not confirmed sepsis (false positive alert). Total of *n* = 440 false positive sepsis alerts. A patient may have had more than one medical condition.

Medical condition	*N* (%)
Gastrointestinal	115 (26.1)
Trauma	113 (25.7)
Cardiovascular	88 (20.0)
Respiratory	43 (9.8)
Overdose/intoxication	42 (9.6)
Central nervous system	39 (8.9)
Renal	34 (7.7)
Hematologic–Oncologic	15 (3.4)
Other	119 (27.1)

Of the 300 randomly selected non-sepsis alert patients, none exhibited sepsis on manual chart review (estimated negative predictive value 100.0%; 95% CI [98.8–100.0%]). The true negative non-sepsis alerts included a range of patients with infections that did not fulfill SIRS criteria (Table 4).

**Table 4:** Medical conditions of emergency department visits without triggered sepsis alert and without confirmed sepsis (true negative alerts). Sample includes a total of *n* = 300 patients not triggering a sepsis alert. A patient may have had more than one medical condition.

Medical condition	*N* (%)
Urinary tract infections	27 (9.0)
Respiratory infections	25 (8.3)
Abscess	8 (2.7)
Cellulitis	5 (1.7)
Gastrointestinal infections	4 (1.3)
Gynecologic infections	4 (1.3)
CNS infections	0 (0.0)
Bacteremia	0 (0.0)
Other infections	21 (7.0)
Trauma	42 (14.0)
Non-infection gastrointestinal conditions	30 (10)
Non-infection CNS	16 (5.3)
Drug overdose	11 (3.7)
Cardiovascular conditions	8 (2.7)
Non-infection respiratory	3 (1.0)
Non-infection renal	4 (1.3)
Hematologic–Oncologic	1 (0.3)
Non-infection other	136 (45.3)

Among true-positive sepsis alert patients, over 90% were admitted to the hospital or died in the ED (Table 5). False-positive sepsis alert patients also exhibited high rates of hospital admission or ED death. Few non-sepsis alert patients were admitted to the hospital.

**Table 5:** Emergency department disposition of true-positive sepsis alert, false-positive sepsis alert, and non-sepsis alert patients.

Emergency department disposition	Type of sepsis alert		
	True-positive sepsis alert *N* (%)	False-positive sepsis alert *N* (%)	No sepsis alert *N* (%)
Admitted to hospital	323 (91.0)	365 (83.0)	17 (5.7)
Died in ED	1 (0.3)	1 (0.2)	0 (0.0)
Discharged from ED	31 (8.7)	74 (16.8)	283 (94.3)

## Discussion

Over the three-month study period, this novel ED sepsis alert system was activated 795 times, identifying nearly 300 confirmed sepsis cases. Our results suggest that an EMR-based sepsis alert system could be used to identify sepsis patients in the ED.

The number of false positive sepsis alerts in this series is not clinically excessive. The clinical identification of sepsis is extremely difficult, requiring assimilation of clinical, physiologic and laboratory data (Jaimes et al., 2003). Anecdotal data suggest that clinicians often under-detect sepsis cases. Jones & Kline (2005) found that in a survey of emergency medicine physicians at 30 academic tertiary care hospitals, only 7% reported implementing early goal-directed therapy for sepsis, and the primary reason for this low rate was due to the poor identification of sepsis. Other studies have shown that automated detection of medical conditions like abdominal aortic aneurysm and central line-associated blood stream infections is more effective than by manual surveillance alone (Padberg et al., 2009; Woeltje et al., 2011). Our observations indicate that almost one in two sepsis alerts will be associated with a true sepsis case. Thus, the system offers aid in the identification of sepsis cases but with only a modest number of false positives. While we could not formally calculate the sensitivity of the system, the random sample of non-alert patients resulted in no sepsis cases, suggesting that the prevalence of false-negatives (undetected sepsis) may be low.

The number of false-positive sepsis alerts is not surprising given that many non-infectious medical conditions can present with vital signs and laboratory abnormalities that fulfill SIRS crtieria. For example, patients with cardiovascular, respiratory and even toxicologic conditions may present with tachycardia, tachypnea, or leukocytosis. Patients with trauma may exhibit tachypnea and tachycardia secondary to pain. Elevated lactic acid may be present in a range of conditions due to tissue hypoxia and subsequent anaerobic metabolism (Bakker et al., 1996).

However, a notable observation was the high proportion of hospital admissions (>80%) among the false-positive sepsis alerts, which was similar to that of the true-positive alerts. This finding suggests that the majority of cases activating a sepsis alert were high acuity patients. Therefore, the sepsis detection system may in fact have broader applicability as a general indicator of ED patient acuity. Further study is needed to characterize this latter population and to better delineate how the information might be integrated into ED clinical practice.

Prior studies have evaluated the use of EMR clinical decision tools to identify sepsis. Nelson et al. (2011) evaluated the use of an automated surveillance algorithm at the University of Michigan Hospital, classifying sepsis as individuals with ≥2 SIRS criteria plus systolic blood pressure of ≤90. The system demonstrated a sensitivity of 64%, PPV of 54%, and NPV of 99% for detecting severe sepsis with signs of organ dysfunction. Our study enhanced the Nelson et al. (2011) criteria by adding elevated lactate (≥2.0 mg/dL) as an additional inclusion criterion. As expected, this strategy increased the number of detected sepsis cases but at the cost of additional false positives (decreased PPV). Also, the Nelson study was based upon only 1 week of ED visits. Our study included a broader range of ED patients from a 3-month time frame.

Variations of the studied sepsis detection system have been developed for the inpatient setting, incorporating additional laboratory values such as coagulation and hepatic function panels. For this effort, we resisted using these extra values because of the likely increase in the number of false positive sepsis alerts. Also, the combination of SIRS criteria with hypotension or lactate is a widely recognized and accepted paradigm in Emergency Medicine; we believed that the selection of additional variables would introduce confusion in clinical application. We believe that the most important strategy for improving the system’s accuracy is to incorporate automated methods for identifying infections. Currently, the presence of infection is dependent upon clinician interpretation and documentation. Biomarkers such as procalcitonin may potentially complement sepsis detection efforts; a recent study demonstrated that procalcitonin had an excellent NPV (96%) and good sensitivity (75%) and specificity (71%) for identifying bacteremia and pneumonia (Albrich & Mueller, 2011; Torres et al., 2012). Future studies must evaluate these and other strategies.

## Limitations

Due to logistical limitations, we were not able to examine all non-alert ED patients; as discussed previously, this would have required manual review of 18,000 records. However, our comparison with randomly selected controls offered important insights, including the low rates of false negatives. Examination of a larger series would likely have affirmed a higher NPV. The EMR system depended on manual input of vital signs by ED personnel. Delayed or erroneous entries may have altered alert activation patterns. Because of the low number of pediatric patients, we did not study modified sepsis criteria for children (Goldstein, Giroir & Randolph, 2005). We focused on sepsis presenting to the ED setting—not sepsis developing later in the hospital course.

This study also examined the accuracy of automated sepsis detection but not its clinical implementation. ED personnel reaction to sepsis alert data was not an *a priori* objective of this study but is clearly an extremely important factor that merits additional study. An important future study is to determine how activated prompts from the decision support system to the clinician may increase the number of recognized sepsis cases in clinical practice.

## Conclusion

This ED EMR clinical support system identified patients presenting to the ED with sepsis. Automated EMR sepsis detection may provide a viable strategy for ED sepsis identification.

## Supplemental Information

10.7717/peerj.343/supp-1 Supplemental Information 1 Raw data

## References

[ref-1] Albrich WC, Mueller B (2011). Predicting bacteremia by procalcitonin levels in patients evaluated for sepsis in the emergency department. Expert Review of Anti-infective Therapy.

[ref-2] Angus DC, Linde-Zwirble WT, Lidicker J, Clermont G, Carcillo J, Pinsky MR (2001). Epidemiology of severe sepsis in the United States: analysis of incidence, outcome, and associated costs of care. Critical Care Medicine.

[ref-3] Annane D, Bellissant E, Cavaillon JM (2005). Septic shock. Lancet.

[ref-4] Bakker J, Gris P, Coffernils M, Kahn RJ, Vincent JL (1996). Serial blood lactate levels can predict the development of multiple organ failure following septic shock. American Journal of Surgery.

[ref-5] Goldstein B, Giroir B, Randolph A (2005). International pediatric sepsis consensus conference: definitions for sepsis and organ dysfunction in pediatrics. Pediatric Critical Care Medicine.

[ref-6] Jaimes F, Garces J, Cuervo J, Ramirez F, Ramirez J, Vargas A, Quintero C, Ochoa J, Tandioy F, Zapata L, Estrada J, Yepes M, Leal H (2003). The systemic inflammatory response syndrome (SIRS) to identify infected patients in the emergency room. Intensive Care Medicine.

[ref-7] Jones AE (2006). Evidence-based therapies for sepsis care in the emergency department: striking a balance between feasibility and necessity. Academic Emergency Medicine.

[ref-8] Jones AE, Kline JA (2005). Use of goal-directed therapy for severe sepsis and septic shock in academic emergency departments. Critical Care Medicine.

[ref-9] Levy MM, Fink MP, Marshall JC, Abraham E, Angus D, Cook D, Cohen J, Opal SM, Vincent JL, Ramsay G (2003). 2001 SCCM/ESICM/ACCP/ATS/SIS international sepsis definitions conference. Intensive Care Medicine.

[ref-10] Levy S, Heyes B (2012). Information systems that support effective clinical decision making. Nursing Management.

[ref-11] Nelson JL, Smith BL, Jared JD, Younger JG (2011). Prospective trial of real-time electronic surveillance to expedite early care of severe sepsis. Annals of Emergency Medicine.

[ref-12] Padberg FT, Hauck K, Mercer RG, Lal BK, Pappas PJ (2009). Screening for abdominal aortic aneurysm with electronic clinical reminders. American Journal of Surgery.

[ref-13] Rivers E, Nguyen B, Havstad S, Ressler J, Muzzin A, Knoblich B, Peterson E, Tomlanovich M (2001). Early goal-directed therapy in the treatment of severe sepsis and septic shock. New England Journal of Medicine.

[ref-14] Torres A, Ramirez P, Montull B, Menendez R (2012). Biomarkers and community-acquired pneumonia: tailoring management with biological data. Seminars in Respiratory and Critical Care Medicine.

[ref-15] Wang HE, Shapiro NI, Angus DC, Yealy DM (2007). National estimates of severe sepsis in United States emergency departments. Critical Care Medicine.

[ref-16] Woeltje KF, McMullen KM, Butler AM, Goris AJ, Doherty JA (2011). Electronic surveillance for healthcare-associated central line-associated bloodstream infections outside the intensive care unit. Infection Control and Hospital Epidemiology.

